# Dupilumab for bullous pemphigoid: Umbrella review of systematic reviews and meta-analyses

**DOI:** 10.1016/j.jdin.2025.10.004

**Published:** 2025-10-18

**Authors:** Mishek Thapa, Brianna Green, Michael Kasperkiewicz

**Affiliations:** Division of Dermatology, Department of Medicine, David Geffen School of Medicine at University of California Los Angeles, Los Angeles, California

**Keywords:** autoantibody, autoimmune blistering disease, bullous pemphigoid, dupilumab, immunobullous disease, umbrella review

*To the Editor:* The use of dupilumab in bullous pemphigoid (BP) has been increasingly reported,^1^ and the drug was recently approved for its treatment by the Food and Drug Administration.^2^ This umbrella review aimed to determine (1) the quality of systematic reviews/meta-analyses about dupilumab treatment for BP and (2) the evidence for efficacy and safety of this interleukin-4/interleukin-13 inhibitor in these patients.

This umbrella review was conducted following PRISMA guidelines. Literature from the inception of the database until 30 June 2025 was explored using PubMed and the keywords “bullous pemphigoid”, “dupilumab”, and “systematic review”. Eligible studies were peer-reviewed, English language systematic reviews (with or without meta-analyses) about dupilumab-treated BP patients (>1). We excluded nonsystematic reviews, guidelines, basic research studies, and articles not meeting the inclusion criteria. Methodological quality was assessed using the AMSTAR2 tool.

We included 8 suitable systematic reviews including 3 with corresponding meta-analyses published between 2022 and 2025 (Fig 1). These reviews summarized 121 studies (primarily cohort studies and case reports/series) with 939 dupilumab-treated BP patients, but extensive overlap was observed comprising 94 redundant reports. Unique (ie, nonoverlapping) reports were 25 studies with 240 dupilumab-treated BP patients (137 males, 87 females, and 16 nonspecified sex; mean age 72 years). Most systematic reviews were classified as critically low quality according to AMSTAR2. All systematic reviews concluded that dupilumab is an effective and/or safe treatment choice for BP (Table I). To synthesize underrepresented evidence not captured in prior pooled analyses, we conducted a single-arm meta-analysis of 6 of the nonoverlapping studies. Analysis was restricted to studies with ≥9 patients each, comprising a total of 207 patients, to enhance statistical stability while minimizing variability from smaller series where outcome estimates can fluctuate widely. The studies varied in design (3 retrospective cohorts, 3 case series), geographic origin (4 from China, 2 from US), and patient populations (including both idiopathic and immune checkpoint inhibitor-induced BP). A DerSimonian-Laird random-effects model with logit transformation and continuity transformation was applied. Dupilumab achieved a pooled complete response rate of 54.8% (95% CI: 36.6% to 71.8%), partial response rate of 31.7% (95% CI: 16.9% to 51.7%), and combined response rate of 86.5% (95% CI: 80.9% to 90.6%). Substantial heterogeneity was observed for individual outcomes (complete response I^2^ = 71.9%, *P* = .003; partial response I^2^ = 69.6%, *P* = .006), reflecting variation in treatment outcomes across studies. In contrast, the combined response analysis showed no heterogeneity (I^2^ = 0.0%, *P* = .69), indicating consistent overall therapeutic efficacy despite varying distributions of complete versus partial responses.

**Fig 1:**
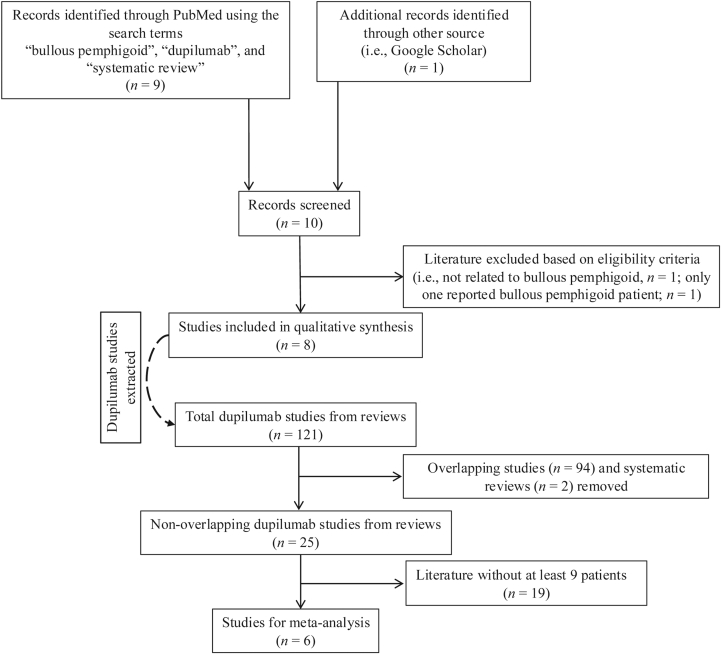
Flowchart of the article selection process.

**Table I:** Characteristics of the included articles pertaining to dupilumab-treated BP patients

Year of publi-cation∗	Country	Journal	Meta-analysis	Databases searched	Search duration	No. of included studies	Individual study design	No. of non-over-lapping studies	No. of over-lapping studies	No. of all dupilumab-treated BP patients	No. of non-overlapping dupilumab-treated BP patients	Outcomes measured	Main results	Main conclusions	AMSTAR2
2025	Canada	Acta Dermato-Venereologica	Yes	MEDLINE, Embase	Inception-Oct 2024	5	Retro-spective cohort (4), pro-spective cohort (1)	0	5	73	0	Time to disease control, predictors of response, achievement of disease control, disease recurrence, adverse events	Dupilumab was associated with significantly shorter time to disease control compared with control	Dupilumab reduces time to disease control in BP	H
2025	Greece	International Journal of Dermatology	No	MEDLINE/PubMed, Web of Science, Scopus	Inception-Jan 2025	13	Retro-spective cohort (3), case reports (7), case series (3)	7	6	43	27	Clinical effectiveness (complete, partial), drug safety	Complete/partial responses were achieved by 93% of dupilumab-treated patients	Dupilumab is highly effective in immune checkpoint inhibitor-induced BP, with most patients achieving complete or partial responses, and has an acceptable safety profile	CL
2025	Brazil	Anais Brasileiros de Dermatologia	Yes	PubMed, Embase, Cochrane Library	Inception-Dec 2023	4	Retro-spective cohort (2), non-randomized trial (1), prospective cohort (1)	0	4	53	0	Time to stop new blister formation, BPDAI, Numeric Rating Scale for pruritus, time to taper methylprednisolone, cumulative and maintenance methylprednisolone dosage, adverse outcomes, relapse	Dupilumab decreased the time before new blister formation stopped (MD = −5.13 d) and demonstrated reduction in BPDAI (MD = −3.90) and Numeric Rating Scale for pruritus (SMD = −1.37). Adverse events and relapses showed no significance	Compared with conventional therapy, dupilumab decreases the time before new blister formation stops as well as BPDAI and pruritus without interfering with adverse events and relapse	CL
2024	Spain	Journal of Clinical Medicine	No	PubMed	Inception-Jan 2024	34	Retro-spective cohort (10), case series (8), case reports (16)	18	16	523	213	Rate of complete response, time to achieve improvement, adverse events	Majority (70.39%) had significant improvement in symptomatology, being a very safe drug with minimal side effects, achieving complete response rates and healing between 2 wk and 6 mo after starting treatment	Dupilumab appears to be an effective option for treating BP in patients refractory to other pharmacological therapies with a good safety profile	CL
2024	China	Dermatologic Therapy	No	PubMed, Embase, Web of Science, Cochrane Library	Inception-Mar 2023	26	Retro-spective study (5), case series (2), case reports (18), mono-centric real-life study (1)	0	26	96	0	Clinical remission (primary), recurrence and adverse events (secondary)	66.7% of patients achieved complete remission, 25.0% had partial remission, 5.2% showed no remission, and no patients experienced deterioration. Average remission time was 4.5 mo. 2 patients relapsed at 8 and 7 mo, respectively. Adverse event rate was 16.9%, of which transient eosinophilia was most common	Dupilumab is a promising treatment for BP with high clinical benefit associated with low recurrence rate, adverse event rate, and mortality	CL
2023	China	International Journal of Derma-tology	Yes	PubMed, Embase, Web of Science, Cochrane Library	Inception-Sep 2022	3	Cohort (3)	0	3	34	0	Short-term effectiveness, adverse events, relapse, long-term survival, BPDAI	Pooled risk ratio for effectiveness in the dupilumab subgroup was not statistically significant	Compared with conventional therapy, dupilumab is associated with fewer adverse events, but no significant difference in efficacy and relapse	L
2023	Germany	Biomolecues	No	PubMed/MEDLINE, Web of Science, Cochrane Library, Scopus, ClinicalTrials.gov	Inception-Jan 2023	23	Retro-spective cohort (2), case series (5), case reports (16)	0	23	81	0	Clinical response (remission, partial, none), time to disease control, relief of pruritus, BPDAI, improvement of skin lesion size and thickness	Two retrospective cohort studies found shorter median time to disease control, more rapid decline of itch, higher quality of life, as well as lower cumulative doses of steroids	As a drug with favorable safety profile as well as established and efficient clinical use for a broad range of patient populations, dupilumab holds significant promise for amending treatment options for a plethora of dermatologic conditions including BP	CL
2022	China	Frontiers in Immunology	No	PubMed, Embase, Web of Science, Cochrane library	Inception-Mar 2022	11	Retro-spective studies (2), case series or reports (9)	0	11	36	0	Complete remission, partial remission, no remission, deterioration, time to remission, recurrence, adverse events	Dupilumab led to complete remission in 66.7% and partial remission in 19.4% of patients within 4.5 mo of treatment without any reported adverse events and a recurrence rate of 5.6%	Dupilumab has similar clinical benefits for BP patients like rituximab and omalizumab, but rituximab results in higher recurrence rates, adverse events, and mortality rates	CL

*BP*, Bullous pemphigoid; *BPDAI*, Bullous Pemphigoid Disease Area Index; *CL*, critically low; *H*, high; *L*, low.

∗ According to the journal's policy, references of the respective publications cannot be displayed because of the limitation in citation numbers.

While our data support the notion that dupilumab is a valuable therapy for BP, the redundancy and very low quality of systematic reviews suggests that results should be interpreted with caution. Future research in this field would benefit from more rigorous qualitative/quantitative synthesis studies. Our results and the recent Food and Drug Administration approval of dupilumab for BP treatment warrant further updated expert consensus recommendations to guide optimal management of patient with BP.^3^

## Conflicts of interest

None disclosed.
